# Predictive Models for Maximum Recommended Therapeutic Dose of Antiretroviral Drugs

**DOI:** 10.1155/2012/469769

**Published:** 2012-02-28

**Authors:** Michael Lee Branham, Edward A. Ross, Thirumala Govender

**Affiliations:** ^1^School of Pharmacy and Pharmacology, University of KwaZulu-Natal, Durban 4001, South Africa; ^2^School of Medicine, University of Florida, Gainesville, FL 32601, USA

## Abstract

A novel method for predicting maximum recommended therapeutic dose (MRTD) is presented using quantitative structure property relationships (QSPRs) and artificial neural networks (ANNs). MRTD data of 31 structurally diverse Antiretroviral drugs (ARVs) were collected from FDA MRTD Database or package inserts. Molecular property descriptors of each compound, that is, molecular mass, aqueous solubility, lipophilicity, biotransformation half life, oxidation half life, and biodegradation probability were calculated from their SMILES codes. A training set (*n* = 23) was used to construct multiple linear regression and back propagation neural network models. The models were validated using an external test set (*n* = 8) which demonstrated that MRTD values may be predicted with reasonable accuracy. Model predictability was described by root mean squared errors (RMSEs), Kendall's correlation coefficients (tau), *P*-values, and Bland Altman plots for method comparisons. MRTD was predicted by a 6-3-1 neural network model (RMSE = 13.67, tau = 0.643, *P* = 0.035) more accurately than by the multiple linear regression (RMSE = 27.27, tau = 0.714, *P* = 0.019) model. Both models illustrated a moderate correlation between aqueous solubility of antiretroviral drugs and maximum therapeutic dose. MRTD prediction may assist in the design of safer, more effective treatments for HIV infection.

## 1. Introduction

Acquired immunodeficiency syndrome (AIDS) is a degenerative disease of the immune and central nervous systems caused by the human immunodeficiency virus (HIV). There are an estimated 33.2 million people living with HIV/AIDS globally [1–3]. Of this number, 22.5 million are in Sub-Saharan Africa, which represents 67.8% of the global number [3]. Antiretroviral drugs (ARVs) may be classified as nucleoside reverse transcriptase inhibitors (NRTIs), nucleotide reverse transcriptase inhibitors (NtRTI), non-nucleoside reverse transcriptase inhibitors (NNRTIs), protease inhibitors (PI), and more recently as fusion or integrase inhibitors [4, 5]. Since most ARVs have low aqueous solubility and poor bioavailability, several alternative drug delivery strategies have been proposed to optimize systemic concentrations [6, 7]. The important biopharmaceutical properties that need to be considered for effective ARV delivery systems might include solubility, pKa, lipophilicity, permeability, stability in biological fluids, gastrointestinal metabolism, and where possible viral reservoir targeting [6, 8, 9]. To overcome suboptimal biopharmaceutical properties, ARVs are often prescribed at high daily doses which increase the occurrence of adverse side effects and toxicities [10, 11]. Combination therapy, comprising at least three anti-HIV drugs, has become a standard treatment of AIDS [12], but here again the potential for adverse side effects and drug-related noncompliance increases. To address these issues, computational methods have been used to predict dose-limiting toxicities of a few antiretroviral drugs [13, 14] or to optimize ARV formulations [15, 16]. The ability to predict maximum therapeutic dose directly from molecular structure is both clinically and scientifically attractive in terms of treatment management and reducing drug development costs [17]. Unfortunately, such models for drugs used in the treatment of AIDS do not yet exist. Accurate prediction of the MRTD for antiretroviral type compounds would be particularly useful in formulation studies so that clinically relevant extrapolations on drug dissolution and permeability can be made earlier in the drug development process [17–20]. Several recent studies have been conducted to define a relationship between the dose and physicochemical properties of the drug [20, 21], or to investigate the underlying mechanisms of drug toxicity and bioaccumulation [20, 22]. Still, the prediction of optimal dose continues to challenge pharmaceutical scientists because of its complexity and variability between different organisms. Artificial neural networks (ANNs) have emerged as a powerful tool suitable for processing complex relationships between molecular stimuli and biological system responses [23]. Examples include prediction of warfarin maintenance dose [24], gentamicin steady-state plasma concentrations [25], skin permeability [26], and prediction of HIV drug resistance [27]; supporting data for these and other studies suggest the utility of neural network modeling for predicting maximum therapeutic doses. 

We believed that since the MRTD estimates are derived from human data, they would provide a more relevant, accurate, and specific estimate for toxic dose levels compared to risk assessment models based on animal data alone. In this article, we predict the MRTD of antiretroviral drugs from their molecular structures using relevant molecular property descriptors and neural network software as a data mining tool. Predictive performance of the models were evaluated and statistically compared with the results obtained clinically or reported in the literature. The application of predictive models in the design of safe, effective antiretroviral drug delivery systems is discussed.

## 2. Materials and Methods

### 2.1. Chemoinformatic Software and Modeling Tools

The physicochemical descriptors, molecular weight (MW), aqueous solubility (ASol), and lipophilicity (AlogP) were determined using ALOGPS 2.1. Virtual Computational Chemistry Laboratory, (http://www.vcclab.org/) [28, 29]. Bioaccumulation descriptors, log biotransformation half life (logBioHL), oxidation half life (OxidHL), and biodegradation probability (P[BD]) were determined using EPI Suite v.410 (http://www.epa.gov/oppt/sf/tools/methods.htm) [30, 31]. All inferential statistics and MRTD data analysis was performed using MedCalc v.12 (MedCalc Software bvba, Belgium). Artificial neural network analysis was performed using Tiberius Data Mining Software v.6.1.9 (Tiberius Data Mining Software Ltd. Pty, UK). 

### 2.2. MRTD Training and Validation Datasets

The MRTD of 31 structurally diverse antiretroviral drugs were taken from the FDA MRTD database or package inserts. This “clinical MRTD” dataset was randomly split into training and validation subsets as shown in Table 1. Subsequently, each of the calculated descriptors and clinical MRTD values were correlated by multiple linear regression analysis and the results used to identify statistically significant property descriptors. An error back propagation algorithm was used for network training, with learning rate set at 0.7. A tangent sigmoid transfer function on the first layer and two neurons with a nonlinear transfer function on the hidden layer were minimalistic structures used to reduce over-fitting. Results of MRTD versus molecular property descriptors with multiple correlation coefficients are listed in Table 2. For the neural network model training, correlation and error statistics are listed in Table 3.

### 2.3. Model Validation and Statistical Comparisons

The predictability of each of the multiple linear regression (MLR) and neural network (TNN) models was evaluated by a cross-validation procedure [32]. Each model was constructed on the basis of the same training dataset and was subsequently used to predict the excluded test data. Statistical comparisons were performed between the clinical MRTD and predicted MRTD values using the root mean squared error (RMSE), Kendall's correlation coefficient (tau), *P*-value, and Bland Altman plots for methods comparisons. 

## 3. Results

### 3.1. Model Predicted versus Actual MRTD Estimates

**Figure 1:**
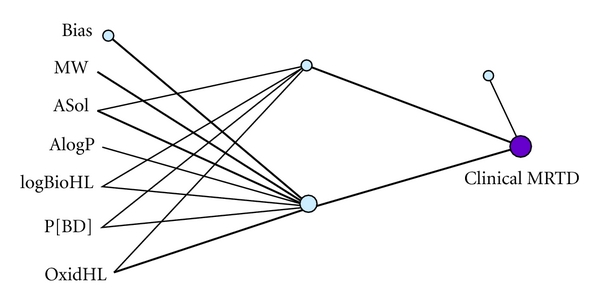
A neural network model was constructed with (2) hidden neuron, (1) output variable that is Clinical MRTD, and (6) input variables that is, OxidHL, P[BD], logBioHL, AlogP, ASol, MW. Total number of patterns (23) were loaded in the data of which 23 were complete and available for training. Two nonlinear neurons were used and the model error minimization was stable for 20 minutes.

In this multivariable system, a quantitative relationship between certain molecular property descriptors and maximum recommended therapeutic dose was characterized using two datasets (i.e., MRTD and TEST). A multiple linear regression (Table 2) and a 6-2-1-neural network model (Figure 1) for the prediction ARVs maximum dose were constructed. Table 1 lists summary statistics for the molecular property descriptors and corresponding MRTD values in each dataset. The ARVs in this study, although few in number, covered a broad range (CV equal to 40% or greater) in terms of the physicochemical (MW, ASol, AlogP) and bioaccumulation properties (logBioHL, OxidHL, P[BD]) included. A significant difference in mean ASol between training set (6.1489 g/L) and TEST set (0.436 g/L) was noted although corresponding changes in lipophilic character were not as great. Multiple linear regression analysis was performed with results listed in Table 2. A statistically significant but modest correlation between two of the six descriptors, that is, P[BD] (*R*_*z*_ = 0.427, *P* = 0.035), ASol (*R*_*z*_ = 0.476, *P* = 0.014) and actual MRTD value is noted. MRTD values appear to increase with P[BD], ASol, and OxidHL; but decrease with increasing logBioHL, AlogP, or MW. The multiple correlation coefficient (MCC = 0.7727), residual error (RSD = 33.89), and ANOVA (*P* = 0.013) indicate acceptable predictability of the multiple regression model for ARVs maximum dose. A multiple linear regression equation used for the prediction of MRTD is as follows:

(1) $$\begin{array}{c} -34.3303-12.20*\left(\text{AlogP}\right)+2.1249*\left(\text{ASol}\right)\\ +15.90*\left(\text{logBioHL}\right)+0.2159*\left(\text{MW}\right)\\ \;+5.659*\left(\text{OxidHL}\right)+46.5133*\left(\text{P[BD]}\right). \end{array}$$

The results of the neural network model for antiretroviral MRTDs is shown in Table 3. All molecular descriptors show weak correlation with MRTD except ASol, OxidHL, and P[BD]. The 6-2-1 neural network predicted training set MRTD values with high accuracy (*R* ^2^ = 0.992, MAX = 13.64, *P* < 0.001). No multicollinearity between independent variables was observed in the training set. SPSS code for the 6-2-1 neural network may be executed as follows:

COMPUTE Var1 = ((OxidHL * (−0.543086558961242)) + 3.63976611815824)
+ ((Pr_BD_ * (−5.67413921537257)) + 2.81153598121711)
+ ((LogBioHL * (0.40828801067156)) + 0.747514104338025)
+ ((ALogP * (−0.848110602763694)) + 1.43330691867064)
+ ((Sol * (−2.55399028293542E−02)) + 0.517313285798853)
+ ((MW * (1.40343495376914E−02)) − 5.94322423917294)
+ 3.86733992096867.
COMPUTE Var2 = ((OxidHL * (8.23491975851945E−02)) - 0.551904322215974)
+ ((Pr_BD_ * (3.12529587401663)) - 1.54858410557524)
+ ((LogBioHL * (0.431493610228662)) + 0.790000076287146)
+ ((ALogP * (−0.04107624611641)) + 6.94188559367329E−02)
+ ((Sol * (4.12997852313067E−02)) − 0.836531279838639)
+ ((MW * (1.05078568602304E−03)) − 0.444983569959981)
+ 9.28741349960935E-02.
COMPUTE Var3 = −0.535249116383622.
Execute.
COMPUTE Var1 = 0.108990429907616 * Var1.
COMPUTE Var2 = 1.44376333322051 * (Exp(Var2) − Exp(-Var2)) / (Exp(Var2) + Exp(−Var2)).
COMPUTE Var3 = Var3.
Execute.
COMPUTE Tiberius_MRTD = ((((Var1 + Var2 + Var3)/2.0) + 0.5) * 199.9625) + 0.0375.
Execute.

### 3.2. Model Validation and Statistical Comparisons

**Figure 2:**
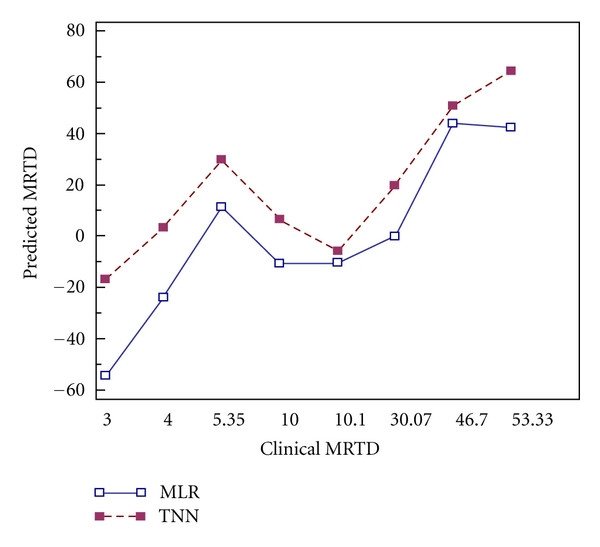
Method comparisons graph. MRTD values predicted by multiple linear regression (hollow squares, □) nearly traced those values predicted by the 6-2-1 neural network (solid squares, ■) as shown. Multiple linear regression estimates for MRTD were consistently lower that those predicted by the neural network model.

**Figure 3:**
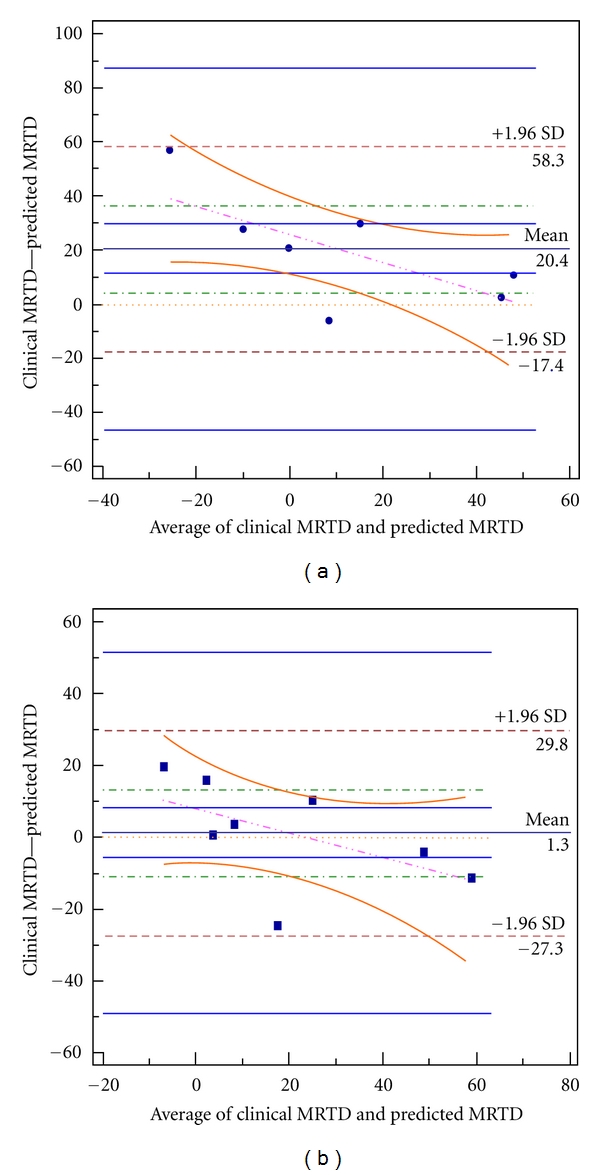
Bland Altman plot for method comparisons. Bland Altman plots are shown for (a) multiple linear regression predicted versus clinical MRTD, and (b) neural network model predicted versus clinical MRTD. Horizontal lines are drawn at the mean difference, and at the limits of agreement. All predicted values were within limits of agreement for both models, althought these limits were more narrow for the neural network model estimates. The plots are useful revealing the relationship between the differences and the averages, the slight deviation from symmetry in the multiple regression indicates some systematic bias but no possible outliers were identified.

Each of the models was then validated using external TEST MRTD dataset and a cross-validation procedure. Model “goodness of fit” and predictability are summarized in Table 4. RMSE of the 6-2-1 neural network (RMSE = 13.67) was substantially less the multiple linear regression model (RMSE = 27.27). Comparison of model predictivity was confirmed with TNN having maximum squared error (SE = 601.23) compared to MLR (SE = 3270.26). Model Figure 3 correlation with the clinical MRTD values using Kendall's correlation coefficient was, however, greater for the MLR (tau = 0.714, *P* = 0.019) than the resultant 6-2-1 neural network model (tau = 0.643, *P* = 0.035). Bland Altman plots for methods comparison further illustrate the predictive value of the neural network (TNN) and multiple linear regression (MLR) models. The upper and lower limits of agreement for the MLR (58.3, −17.4) and TNN (29.8, −27.3) are illustrated in Figure 2. While it can be seen that all of the differences lie between these limits, MLR model limits of agreement were wider than the TNN model, which were more narrow and nearly symmetrical.

## 4. Discussion

MRTD values and SMILES codes for antiretroviral drugs were collected from the FDA MRTD database which is a highly reliable source pharmacologic activity based on extensive clinical evidence (http://www.fda.gov/cder/). The ALOGPS+ program was used for the calculation of molecular mass (MW), solubility (ASol), and lipophilicity (AlogP) without alteration. The precision and robustness of these tools are well established in the pharmaceutical and modeling community and its predictive power concerning the input parameters in question are published on the company website (http://www.vcclab.com/) and cited in the manuscript [28, 29, 33]. Independent validation of the parameters calculated with ALOGPS has been published [33, 34] and the estimates were found to be appropriately accurate for the use intended. The bioaccumulation input parameters, oxidation half life (oxidHL), log biotransformation half life (logBioHL), and biodegradation probability (P[BD]) were calculated using the EPI Suite software which is publically available and has been validated in hundreds of modeling experiments for the estimated parameters. EPI Suite software is continuously updated and the predictive power of its latest version is always available at the website (http://www.epa.gov/oppt/sf/tools/methods.htm). None of the parameters were log-transformations of the software output but instead used as direct input values for the training set. Although some of the predictor values ranged over 3 orders of magnitude, this did not appear to adversely affect comparisons of the MLR or ANN methods. Furthermore, because of the highly diverse nature of antiretroviral compounds in terms of physicochemical descriptors and split-data validation procedure with randomly selected training and test sets instead of cross-validating scheme. It is our intention to adapt the method as new ARV drugs become listed in evolving FDA database. 

We began our study looking at dose-related adverse effects of commercial antiretroviral drugs or new ARVs in development. Although the appearance of serious long-term metabolic complications, such as cardiovascular disturbances [35], hyperlipidemia [35, 36], and diabetes, have been extensively reported [35, 37], few reports on computational models to predict these dose-limiting toxicities [38, 39] can be found in the literature. A mathematical model to predict the optimal dosing regimen for AIDS therapy has been reported [40], but in this case, CD4+ cell counts and knowledge of the adherence interval of individual patients is required to adjust the dose. While the model was effective at reducing dose-limiting toxicities in an AIDS patient population, it cannot be applied to nonapproved ARV formulations or to drugs in development. Since the overwhelming majority of anti-HIV drugs demonstrate efficacy over a small range of treatment doses, MRTD predicting models would be beneficial to the drug delivery scientist who needs to design experiments based on the most effective therapeutic dose of the medication [41]. The MRTD is empirically derived from human clinical trials, thus it is a direct measure of the threshold for dose-related adverse effects in humans. Prediction of the MRTD from molecular structure is of importance for both new and existing drugs which may require modifications to improve their aqueous solubility or bioavailability *in vivo*. ARVs fit this description also and are excellent subject molecules for predictive modeling to estimate maximum dose. Unfortunately, the number of antiretroviral drugs with established MRTD is yet small, so the development of models specifically of ARVs is both tedious and rare. 

The molecular descriptors used in this study were selected to represent physicochemical (MW, AlogP, ASol) and bioaccumulation (OxidHL, P[BD], logBioHL) property influences therapeutic dose. Although molecular weight does not strongly correlate with toxicity of most compounds, the larger the molecular size of a compound, the smaller its membrane permeability and diffusion coefficient become [34]. Therefore, compounds with higher weights are less likely to be absorbed, which limits their systemic toxicity. Results of this study predict no correlation between molecular weight and maximum ARV dose. In contrast, bioactivity and drug toxicity almost always increase with increasing lipophilicity. This is due in part to the fact that lipophilic molecules tend to cross cellular membranes more readily increasing exposure and residence in the body. In addition, lipophilic drugs are characterized by increased plasma protein binding, thus an assessment of lipophilicity is almost always included in the physicochemical evaluation of a drug because of its close association with pharmacologic, permeability and potential bioaccumulation [42, 43]. Our results here did not indicate AlogP or lipophilic character as having any influence on ARV dose. The aqueous solubility of a compound significantly affects its absorption and distribution characteristics. Typically, low solubility goes along with a poor absorption and, therefore, its systemic toxicity reduced, however, local irritability may develop and/or reduced elimination rates, both are characteristic of drugs with low aqueous solubility. For highly potent drugs, increasing solubility usually enhances the elimination rate and lowers systemic half life, [44]. This means that for ARVs with low aqueous solubility, some correlation with maximum therapeutic dose (as we has shown) is likely to be observed. 

Any chemical (even water) can produce toxic side effects in the body if allowed to accumulate to sufficiently large concentrations. While much of the effort in bioaccumulation modeling [43, 45] has been initiated by scientists estimating the equilibrium distribution of chemicals between organisms and their environments (e.g., fish-water, plants-soil), effective physicochemical property estimation routines (i.e., PERs) have resulted from these studies that may be applied to similar biodistribution problems in pharmaceutical research. For example, oxidation half life (OxidHL) is an estimate of the molecules ability to form stable hydroxyl radicals or to interact with such moiety under ambient conditions. Formation of hydroxyl-radicals is often associated with dose-limiting toxicities. Although in our studies, oxidation half life was not statistically significant in the prediction of MRTD. Another bioaccumulation descriptor, biotransformation half life (logBioHL), is the (linearized) fraction of drug mass in the whole body that has been metabolized per day. Our estimates do not account for the formation of specific metabolites which may be toxic nor do they identify specific pathways in the process (i.e., phase I redox reactions or phase II type conjugation reactions). Consequently, logBioHL was not correlated with MRTD in the present study and was a weaker bioaccumulation property descriptor in comparison to oxidation half life or biodegradation probability P[BD]. The probability of biodegradation attempts to combine both oxidative and biotransformation susceptibility of the structure to give an estimate of overall persistence. P[BD] estimates are based upon molecular fragments [46–48] and in our investigation showed only moderate correlation with MRTD in both multiple linear regression and neural network models. Each of the models was evaluated for predictive accuracy and by statistical comparison. In terms of predictability, root mean squared errors were larger for the multiple linear regression than the neural network model. Some advantages of ANN over MLR models were illustrated in the current study. 

Artificial neural networks (ANNs) are biologically inspired data-mining algorithms which work by detecting the patterns and relationships in data. We used the back propagation rule in which the neural network is trained to map a set of input data by iterative adjustment of the weights. A tangent sigmoid transfer function on the first layer and two neurons with a nonlinear transfer function on the hidden layer were minimalistic structures used to reduce overfitting. Our training processes for TNN were allowed to run until no change in RMSE was observed for 20 minutes, at which point the model was saved. This learning method is commonly used for neural network predictive models given dose-response type data. However, ANNs have several limitations, a major theoretical concern is the “black box” nature of the output, that is, conclusions are generated without mechanistic explanations. ANNs also are limited by the quality of their data and may need to be retrained periodically if its performance changes over time. This is not necessarily counterproductive, since it indicates robustness in the model which adapts to changes in the predictive criteria. Real-time monitoring of the training process is also important since overtraining can easily occur, especially when the datasets are small in size. This is may be one of the unique advantages of real-time visualization of the data-mining process allowing the investigator to make “intermediate evaluations” of model predictability and then continue training until the reliability and accuracy required of the predictions are met.

In conclusion, antiretroviral drugs are a chemically diverse class of compounds in terms of both physicochemical properties and bioaccumulation potential. However, commercial ARVs may be categorized for predictive modeling purposes into two groups based on aqueous solubility and lipophilic character, in which hydrophilic compounds may be administered at higher doses (MRTD) and prediction of their MRTD value may be possible using simple multiple linear regression models. In contrast, the prediction of MRTD values for antiretrovirals with poorer aqueous solubility would be the most effective when the neural network approach is used and when both physicochemical and bioaccumulation property descriptors are available for training. With regard to future studies, ANN represents a promising tool for predicting maximum therapeutic dose, especially for antiretroviral drugs with narrow therapeutic index in the treatment of AIDS.

## Figures and Tables

**Table 1:** Clinical MRTD data consisted of 23 training set compounds (italic) and 8 test set compounds (bold). Mean and standard deviation statistics are shown indicating the distribution and central tendency of each molecular property descriptor.

Drug	MRTD (mg/kg/day)	OxidHL (days)	P[BD] (%)	logBioHL (days)	AlogP (:)	ASol (g/L)	MW (Da)
*Acyclovir*	13.30	0.135	0.5475	−2.2874	−1.45	8.65	225.21
*Ancitabine*	20.00	0.139	0.2005	−2.418	−2.62	3.20	225.21
*Delaviridine*	6.67	0.034	0.0014	−2.3428	2.77	0.086	456.57
*Didanosine*	6.67	0.140	0.5085	−1.9956	−1.26	6.43	236.23
*Famciclovir*	25.00	0.051	0.991	−3.6235	0.13	1.32	321.38
*Foscarnet*	120.00	13.37	0.772	−2.4547	−1.63	16.76	126.01
*Indinavir*	16.70	0.038	0.0501	−4.6478	3.26	0.048	613.81
*Lofexidine*	0.040	0.123	0.0792	0.9861	3.31	0.15	259.14
*Lamivudine*	5.00	0.06	0.0719	−3.9261	−1.29	2.76	229.26
*Rimantadine*	3.33	0.171	0.4624	0.4539	3.28	0.009	179.31
*Ribavirin*	200.00	0.264	0.8963	−2.8715	−1.92	33.17	244.21
*Valacyclovir*	50.00	0.044	0.944	−3.3309	−1.03	1.49	326.41
*Zalcitibine*	0.0375	0.096	0.0909	−3.5083	−1.29	7.05	211.22
*Zanamivir*	0.333	0.038	0.8247	−4.4141	−2.29	1.49	332.32
*Zidovudine*	10.00	0.139	0.0432	−3.0445	−0.1	16.35	267.28
*Saquinavir*	33.330	0.052	0.9780	−3.9394	4.04	0.002	670.84
*Darunavir*	53.00	0.094	0.0001	−1.8175	1.76	0.067	547.66
*Tipranavir*	6.670	0.043	0.0000	−0.0265	5.71	0.0002	602.66
*Ritonavir*	20.00	0.105	0.9488	−4.61	4.24	0.0012	720.94
*Maraviroc*	20.00	0.129	0.0700	−0.2247	4.3	0.0106	513.66
*Tenofovir*	10.00	0.048	0.0016	−3.0098	−1.51	1.87	287.21
*Nelfinavir*	25.00	0.055	0.6868	−1.6709	6.00	0.0002	567.78
*Stavudine*	1.333	0.088	0.0768	−3.082	−0.8	40.51	224.21

*n*	23.00	23.00	23.00	23.00	23.00	23.00	23.00
mean	28.54	0.672	0.402	−2.5133	0.939	6.15	362.72
SD	45.53	2.769	0.394	1.5738	2.809	10.90	178.20
CV%	159.52	412.00	97.00	−62.620	299.04	177.25	48.86

**Abacavir**	10.00	0.040	0.0229	−2.1757	0.61	1.21	286.38
**Emtricitabine**	4.00	0.08	0.0566	−3.7784	−0.8	2.00	247.28
**Raltegravir **	30.07	0.094	0.0006	−2.6157	1.7	0.095	444.47
**Nevirapine**	3.000	0.167	0.0473	−3.7336	1.75	0.10	266.33
**Efavirenz **	10.10	0.254	0.0001	0.1111	3.88	0.008	315.67
**Fosamprenavir **	46.70	0.067	0.0007	−2.4135	0.84	0.068	585.68
**Atazanavir **	5.35	0.109	0.0202	−3.4392	4.37	0.003	704.96
**Lopiravir **	53.33	0.103	0.9933	−3.3281	4.07	0.002	614.86

*n *	8.00	8.00	8.00	8.00	8.00	8.00	8.00
mean	20.32	0.114	0.143	−2.672	2.05	0.436	433.20
SD	20.29	0.067	0.344	1.278	1.878	0.753	180.48
CV%	99.86	58.99	241.300	−47.830	91.50	173.000	41.66

**Table 2:** The results of the multiple linear regression analysis for antiretroviral drugs versus MRTD. In the training, dataset only two of the six molecular descriptors showed a statistically significant correlation with therapeutic dose, that is, P[BD] (*P* = 0.0349) and ASol (*P* = 0.0140). A multiple regression model equation coefficients are listed with there standard errors, coefficient of determination (*R* ^2^ = 0.5970), multiple correlation coefficient (MCC = 0.7727) and residual standard deviation (RSD = 33.89).

Multiple linear regression	*n* = 23	*R* ^2^ = 0.5970	RSD = 33.89	MCC = 0.7727
independent variables	Coefficient	SE	*P*-value	*R*_**z**_
Constant	−34.3303			
AlogP	−12.1995	6.6976	0.0873	−0.221
ASol	2.1239	0.7697	0.0140	0.476
logBioHL	15.9000	8.0377	0.0654	−0.071
MW	0.2159	0.1015	0.0494	−0.116
OxidHL	5.6589	2.8478	0.0643	0.448
P[BD]	46.5133	20.1746	0.0349	0.427

ANOVA	F-ratio = 3.9507	*P* = 0.013		

**Table 3:** The results of the neural network analysis for antiretroviral drugs versus MRTDs. All molecular descriptors show weak correlation with MRTD except for ASol, OxidHL, and P[BD]. The 6-2-1 neural network model predicted training set MRTD values with high accuracy (*R* ^2^ = 0.992, MAX = 13.64, *P* < 0.001). No multicollinearity between independent variables was observed in the training set.

6-2-1 neural network	*n* = 23	*R* ^2^ = 0.992	MAX = 13.64	RMSE = 5.53
Model versus clinical MRTD	*P* < 0.001		Learning rate = 0.700	
Independent variables	Correlation coefficients		*R* ^2^	
AlogP	−0.221		0.049	
ASol	0.476		0.226	
logBioHL	−0.071		0.005	
MW	−0.116		0.013	
OxidHL	0.448		0.201	
P[BD]	0.427		0.182	

ANOVA	F-ratio = 1340.73		*P* < 0.001	

**Table 4:** Test dataset Goodness of fit comparisons. Clinical MRTD values from 8 ARVs were using as and external test dataset the validate model predictability. Model performance is characterized here in terms of root means squared error (RMSE), Kendall's correlation coefficient (tau), and Type II error probability (*P*-value).

Drug	MRTD	MLR	SE	TNN	SE
Abacavir	10.00	−10.6738	427.4060	6.4861	12.3474
Emtricitabine	4.00	−23.9239	779.7441	3.3678	0.3996
Raltegravir	30.07	0.0628	900.4320	19.7700	106.0900
Nevirapine	3.00	−54.1862	3270.2614	−16.5700	382.9849
Efavirenz	10.10	−10.2863	415.6012	−5.7782	252.1172
Fosamprenavir	46.70	44.0515	7.0145	50.7500	16.4025
Atazanavir	5.35	11.4360	37.0393	29.8700	601.2304
Lopiravir	53.33	42.6361	114.3594	64.5900	126.7876

Mean	20.32	−0.1105	RMSE = 27.27	19.06	RMSE = 13.67
Kendall's tau		0.714		0.643	
*P*-value		0.019		0.035	

## Abbreviations

AlogP: Calculated octanaol/water partition coefficient
ASol: Aqueous solubility
logBioHL: Biotransformation half life
CV: Coefficient of variation
*R*^2^: Coefficient of determination
MLR: Multiple linear regression
MRTD: Maximum recommended therapeutic dose
MAX: maximum absolute error
MW: Molecular weight
MCC: Multiple correlation coefficient
OxidHL: Oxidation half life
P[BD]: Biodegradation probability
TNN: Tiberius Neural Network
QSPR: Quantitative Structure Property Relationships
RSD: Residual standard deviation
RMSE: Root mean squared error
SMILES: Simplified molecular input line entry system
SE: Squared error
SD: Standard deviation
*R*_*z*_: Zero order correlation coefficient.
